# Di-μ-hydroxido-bis­[tris­(4,4,4-trifluoro-1-phenyl­acetyl­acetonato-κ^2^ *O*,*O*′)hafnium(IV)] dimethyl­formamide disolvate

**DOI:** 10.1107/S1600536811049543

**Published:** 2011-11-25

**Authors:** J. Augustinus Viljoen, Hendrik G. Visser, Andreas Roodt

**Affiliations:** a Department of Chemistry, University of the Free State, PO Box 339, Bloemfontein 9300, South Africa

## Abstract

The binuclear molecule of the title compound, [Hf_2_(C_10_H_6_F_3_O_2_)_6_(OH)_2_]·2C_3_H_7_NO, lies across an inversion centre and contains a Hf^IV^ atom which is eight-coordinated and surrounded by three chelating β-diketonato tris­(4,4,4-trifluoro-1-phenyl­acetyl­acetonate (tfba^−^) ligands and two bridging OH^−^ groups in a distorted square-anti­prismatic geometry. The Hf—O bond lengths vary from 2.073 (2) to 2.244 (2) Å and the O—Hf—O bite angles vary from 73.49 (9) to 75.60 (9)°. Weak O—H⋯O hydrogen-bonding inter­actions are observed between the bridging hy­droxy groups and the dimethylformamide solvent mol­ecules. The unit cell contains solvent-accessible voids of 131 Å^3^, but the residual electron density in the difference Fourier map suggests no solvent mol­ecule occupies this void.

## Related literature

For our ongoing research investigating the reactions of various *O*,*O*′-and *N*,*O*-bidentate ligands with hafnium(IV) and zirconium(IV) to exploit possible separation techniques and for the crystal structures of hafnium(IV) and zirconium(IV) complexes, see: Viljoen *et al.* (2010 ▶); Steyn *et al.* (2011 ▶).
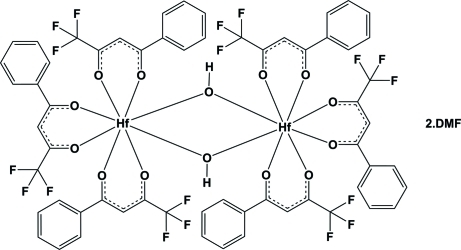


## Experimental

### 

#### Crystal data

[Hf_2_(C_10_H_6_F_3_O_2_)_6_(OH)_2_]·2C_3_H_7_NO
*M* *_r_* = 1828.08
Monoclinic, 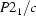

*a* = 12.4143 (3) Å
*b* = 19.244 (5) Å
*c* = 17.503 (5) Å
β = 122.937 (5)°
*V* = 3509 (2) Å^3^
*Z* = 2
Mo *K*α radiation
μ = 3.07 mm^−1^
*T* = 100 K
0.28 × 0.23 × 0.21 mm

#### Data collection

Bruker X8 APEXII 4K KappaCCD diffractometer
Absorption correction: multi-scan (*SADABS*; Bruker, 2004 ▶) *T*_min_ = 0.431, *T*_max_ = 0.526
41903 measured reflections
8726 independent reflections
6864 reflections with *I* > 2σ(*I*)
*R*_int_ = 0.050

#### Refinement

*R*[*F* ^2^ > 2σ(*F* ^2^)] = 0.030
*wR*(*F* ^2^) = 0.072
*S* = 1.03
8726 reflections
475 parameters
1 restraint
H atoms treated by a mixture of independent and constrained refinement
Δρ_max_ = 1.33 e Å^−3^
Δρ_min_ = −0.97 e Å^−3^

### 

Data collection: *APEX2* (Bruker, 2005 ▶); cell refinement: *SAINT-Plus* (Bruker, 2004 ▶); data reduction: *SAINT-Plus*; program(s) used to solve structure: *SIR92* (Altomare *et al.*, 1999 ▶); program(s) used to refine structure: *SHELXL97* (Sheldrick, 2008 ▶); molecular graphics: *DIAMOND* (Brandenburg & Putz, 2005 ▶); software used to prepare material for publication: *WinGX* (Farrugia, 1999 ▶).

## Supplementary Material

Crystal structure: contains datablock(s) I, global. DOI: 10.1107/S1600536811049543/pv2484sup1.cif

Structure factors: contains datablock(s) I. DOI: 10.1107/S1600536811049543/pv2484Isup2.hkl

Additional supplementary materials:  crystallographic information; 3D view; checkCIF report

## Figures and Tables

**Table 1:** Hydrogen-bond geometry (Å, °)

*D*—H⋯*A*	*D*—H	H⋯*A*	*D*⋯*A*	*D*—H⋯*A*
O7—H1*A*⋯O8	0.78 (2)	1.94 (2)	2.712 (3)	171 (5)

**Fig. 1.**
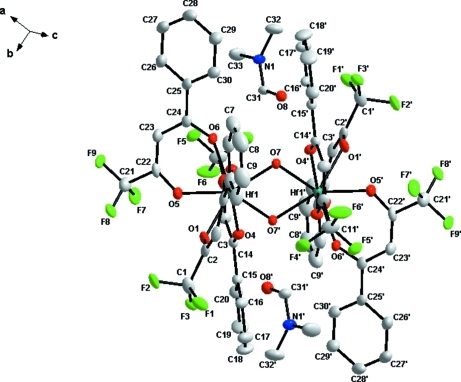
Representation of the title compound, showing the numbering scheme and displacement ellipsoids (50% probability). Primed atoms are related to the unprimed atoms by symmetry operation: -x+1, -y+1, -z+1.

## supplementary crystallographic information

### Comment

This study forms part of ongoing research to investigate the reactions of various O,O'-and N,O-bidentate ligands with hafnium(IV) and zirconium(IV) to exploit possible separation techniques (Steyn *et al.* 2011; Viljoen *et al.* (2010).

The metal complex of the title compound (Figure 1) consists of a Hf^IV^ atom which is eight-coordinated and surrounded by three tfba^-^ ligands and two bridging OH-groups thereby adopting a slightly distorted anti-prismatic coordination geometry. The Hf—O bond lengths vary from 2.073 (2) Å to 2.244 (2) Å and the O—Hf—O bite angles vary from 73.49 (9) ° to 75.60 (9) °. The dimer skeleton exhibits a flat diamond-like structure and lies across an inversion centre with Hf—O7, Hf—O7^i^ and Hf—Hf^i^ distances of 2.135 (2), 2.073 (2) and 3.4958 (8) Å, respectively, and a bite angle of 67.66 (11) °. Lastly, weak hydrogen bonding interactions are observed between one of the bridging hydroxy groups (O7—H1A) and the solvent molecule (Table 1).

### Experimental

Chemicals were purchased from Sigma and Aldrich and used as received. 4,4,4-Trifluoro-1-phenyl-1,3-butanedione,tfbaH, (540 mg, 2.5 mmol) was added slowly to HfCl_4_ (200 mg, 0.624 mmol) in *N*,*N*-dimethylformamide (25 ml). After refluxing for *ca* 12 h, the crude product was filtered and left to stand at room temperature for colorless crystals to form.

### Refinement

The aromatic, methine, and methyl H atoms were placed in geometrically idealized positions (C—H = 0.93–0.98) and constrained to ride on their parent atoms with *U*_iso_(H) = 1.2*U*_eq_(C) for aromatic and methine, and *U*_iso_(H) = 1.5*U*_eq_(C) for methyl H-atoms. The highest residual electron density was located 1.05 Å from H1A and was essentially meaningless.

### Crystal data

**Table:**

[Hf_2_(C_10_H_6_F_3_O_2_)_6_(OH)_2_]·2C_3_H_7_NO	*F*(000) = 1792
*M**_r_* = 1828.08	*D*_x_ = 1.73 Mg m^−^^3^
Monoclinic, *P*2_1_/*c*	Mo *K*α radiation, λ = 0.71069 Å
Hall symbol: -P 2ybc	Cell parameters from 9995 reflections
*a* = 12.4143 (3) Å	θ = 2.9–28.1°
*b* = 19.244 (5) Å	µ = 3.07 mm^−^^1^
*c* = 17.503 (5) Å	*T* = 100 K
β = 122.937 (5)°	Cuboid, colourless
*V* = 3509 (2) Å^3^	0.28 × 0.23 × 0.21 mm
*Z* = 2	

### Data collection

**Table:**

Bruker X8 APEXII 4K KappaCCD diffractometer	8726 independent reflections
Radiation source: fine-focus sealed tube	6864 reflections with *I* > 2σ(*I*)
graphite	*R*_int_ = 0.050
ω and φ scans	θ_max_ = 28.3°, θ_min_ = 3.0°
Absorption correction: multi-scan (*SADABS*; Bruker, 2004)	*h* = −16→16
*T*_min_ = 0.431, *T*_max_ = 0.526	*k* = −25→25
41903 measured reflections	*l* = −23→22

### Refinement

**Table:**

Refinement on *F*^2^	Primary atom site location: structure-invariant direct methods
Least-squares matrix: full	Secondary atom site location: difference Fourier map
*R*[*F*^2^ > 2σ(*F*^2^)] = 0.030	Hydrogen site location: inferred from neighbouring sites
*wR*(*F*^2^) = 0.072	H atoms treated by a mixture of independent and constrained refinement
*S* = 1.03	*w* = 1/[σ^2^(*F*_o_^2^) + (0.0285*P*)^2^ + 4.244*P*] where *P* = (*F*_o_^2^ + 2*F*_c_^2^)/3
8726 reflections	(Δ/σ)_max_ = 0.004
475 parameters	Δρ_max_ = 1.33 e Å^−^^3^
1 restraint	Δρ_min_ = −0.97 e Å^−^^3^
1 constraint	

### Special details

**Table:**

Geometry. All s.u.'s (except the s.u. in the dihedral angle between two l.s. planes) are estimated using the full covariance matrix. The cell s.u.'s are taken into account individually in the estimation of s.u.'s in distances, angles and torsion angles; correlations between s.u.'s in cell parameters are only used when they are defined by crystal symmetry. An approximate (isotropic) treatment of cell s.u.'s is used for estimating s.u.'s involving l.s. planes.
Refinement. Refinement of *F*^2^ against ALL reflections. The weighted *R*-factor *wR* and goodness of fit *S* are based on *F*^2^, conventional *R*-factors *R* are based on *F*, with *F* set to zero for negative *F*^2^. The threshold expression of *F*^2^ > 2σ(*F*^2^) is used only for calculating *R*-factors(gt) *etc*. and is not relevant to the choice of reflections for refinement. *R*-factors based on *F*^2^ are statistically about twice as large as those based on *F*, and *R*- factors based on ALL data will be even larger.

### Fractional atomic coordinates and isotropic or equivalent isotropic displacement parameters (Å^2^)

**Table:**

	*x*	*y*	*z*	*U*_iso_*/*U*_eq_	
C1	0.5472 (4)	0.7683 (2)	0.4852 (3)	0.0300 (9)	
C2	0.5889 (4)	0.69251 (18)	0.5092 (2)	0.0229 (8)	
C3	0.6788 (3)	0.67550 (19)	0.5996 (2)	0.0234 (8)	
H3	0.6964	0.7074	0.6465	0.028*	
C4	0.7430 (3)	0.6123 (2)	0.6218 (2)	0.0235 (8)	
C5	0.8546 (3)	0.5979 (2)	0.7159 (2)	0.0261 (8)	
C6	0.9359 (4)	0.5421 (2)	0.7303 (3)	0.0332 (9)	
H6	0.9175	0.5136	0.6803	0.04*	
C7	1.0431 (4)	0.5276 (2)	0.8162 (3)	0.0403 (11)	
H7	1.0991	0.4906	0.8246	0.048*	
C8	1.0673 (5)	0.5682 (3)	0.8900 (3)	0.0488 (12)	
H8	1.1394	0.5583	0.9492	0.059*	
C9	0.9859 (5)	0.6229 (3)	0.8769 (3)	0.0447 (12)	
H9	1.0019	0.6498	0.9276	0.054*	
C10	0.8804 (4)	0.6388 (2)	0.7898 (3)	0.0344 (10)	
H10	0.8268	0.6772	0.781	0.041*	
C11	0.3785 (4)	0.4208 (2)	0.1663 (3)	0.0335 (10)	
C12	0.3762 (4)	0.47465 (18)	0.2301 (2)	0.0225 (8)	
C13	0.2820 (3)	0.52375 (18)	0.1939 (2)	0.0233 (8)	
H13	0.2233	0.5263	0.1298	0.028*	
C14	0.2716 (3)	0.57057 (17)	0.2511 (2)	0.0174 (7)	
C15	0.1616 (3)	0.61903 (18)	0.2168 (2)	0.0197 (7)	
C16	0.1675 (4)	0.66931 (19)	0.2764 (3)	0.0267 (8)	
H16	0.2405	0.6717	0.337	0.032*	
C17	0.0675 (4)	0.7157 (2)	0.2477 (3)	0.0377 (10)	
H17	0.0722	0.7505	0.2879	0.045*	
C18	−0.0389 (4)	0.7110 (2)	0.1603 (3)	0.0449 (12)	
H18	−0.109	0.7417	0.1412	0.054*	
C19	−0.0453 (4)	0.6628 (2)	0.1004 (3)	0.0420 (11)	
H19	−0.1182	0.6612	0.0397	0.05*	
C20	0.0543 (3)	0.6165 (2)	0.1283 (2)	0.0279 (8)	
H20	0.0496	0.5829	0.0869	0.033*	
C21	0.6419 (4)	0.6077 (2)	0.2317 (3)	0.0332 (9)	
C22	0.6666 (4)	0.56527 (19)	0.3136 (2)	0.0242 (8)	
C23	0.7556 (4)	0.5129 (2)	0.3462 (3)	0.0279 (8)	
H23	0.8017	0.504	0.3182	0.033*	
C24	0.7798 (3)	0.47168 (18)	0.4211 (2)	0.0204 (7)	
C25	0.8870 (3)	0.42024 (18)	0.4640 (2)	0.0210 (7)	
C26	0.9601 (4)	0.4044 (2)	0.4279 (3)	0.0337 (9)	
H26	0.9425	0.4266	0.3738	0.04*	
C27	1.0581 (4)	0.3563 (2)	0.4706 (3)	0.0401 (11)	
H27	1.1081	0.3462	0.4459	0.048*	
C28	1.0843 (4)	0.3230 (2)	0.5477 (3)	0.0366 (10)	
H28	1.151	0.2894	0.5757	0.044*	
C29	1.0127 (4)	0.3387 (2)	0.5849 (3)	0.0318 (9)	
H29	1.031	0.3163	0.639	0.038*	
C30	0.9153 (4)	0.3868 (2)	0.5430 (3)	0.0259 (8)	
H30	0.8666	0.3973	0.5687	0.031*	
C31	0.6169 (4)	0.31446 (19)	0.4081 (3)	0.0264 (8)	
H31	0.5679	0.3489	0.3634	0.032*	
C32	0.7378 (5)	0.2081 (2)	0.4479 (3)	0.0432 (11)	
H32A	0.7447	0.2167	0.5056	0.065*	
H32B	0.8236	0.209	0.4581	0.065*	
H32C	0.6986	0.1625	0.4241	0.065*	
C33	0.6361 (5)	0.2557 (3)	0.2929 (3)	0.0524 (14)	
H33A	0.578	0.293	0.2544	0.079*	
H33B	0.597	0.2106	0.2664	0.079*	
H33C	0.7176	0.2599	0.2967	0.079*	
N1	0.6595 (3)	0.26113 (16)	0.3833 (2)	0.0285 (7)	
O1	0.5438 (2)	0.65377 (12)	0.44087 (15)	0.0214 (5)	
O2	0.7170 (2)	0.56513 (13)	0.56345 (16)	0.0221 (5)	
O3	0.4665 (2)	0.46540 (12)	0.31343 (15)	0.0206 (5)	
O4	0.3564 (2)	0.57330 (12)	0.33550 (15)	0.0195 (5)	
O5	0.5958 (2)	0.58469 (12)	0.34167 (16)	0.0221 (5)	
O6	0.7133 (2)	0.47712 (12)	0.45494 (16)	0.0191 (5)	
O7	0.5352 (2)	0.44652 (12)	0.48937 (16)	0.0171 (5)	
O8	0.6357 (2)	0.32287 (13)	0.48407 (17)	0.0253 (6)	
F1	0.5519 (3)	0.80248 (12)	0.55312 (15)	0.0458 (7)	
F2	0.6313 (3)	0.80097 (12)	0.47024 (18)	0.0461 (7)	
F3	0.4326 (2)	0.77581 (11)	0.41168 (15)	0.0343 (5)	
F4	0.3554 (3)	0.35768 (12)	0.18342 (19)	0.0472 (7)	
F5	0.4918 (2)	0.41859 (13)	0.17586 (17)	0.0420 (6)	
F6	0.2911 (3)	0.43385 (16)	0.07841 (16)	0.0641 (9)	
F7	0.5246 (3)	0.59463 (14)	0.15994 (16)	0.0494 (7)	
F8	0.6486 (3)	0.67553 (13)	0.24721 (17)	0.0448 (6)	
F9	0.7245 (3)	0.59400 (14)	0.20724 (18)	0.0505 (7)	
Hf1	0.547988 (13)	0.538181 (7)	0.432516 (9)	0.01636 (5)	
H1A	0.572 (4)	0.4128 (15)	0.492 (3)	0.039 (14)*	

### Atomic displacement parameters (Å^2^)

**Table:**

	*U*^11^	*U*^22^	*U*^33^	*U*^12^	*U*^13^	*U*^23^
C1	0.042 (2)	0.027 (2)	0.0213 (18)	−0.0132 (18)	0.0173 (18)	−0.0075 (16)
C2	0.026 (2)	0.0212 (18)	0.0303 (19)	−0.0097 (15)	0.0213 (17)	−0.0065 (15)
C3	0.0222 (19)	0.032 (2)	0.0174 (16)	−0.0139 (16)	0.0114 (15)	−0.0107 (15)
C4	0.0195 (18)	0.031 (2)	0.0223 (17)	−0.0119 (16)	0.0130 (15)	−0.0047 (16)
C5	0.0178 (18)	0.040 (2)	0.0160 (17)	−0.0118 (16)	0.0062 (15)	−0.0041 (15)
C6	0.029 (2)	0.039 (2)	0.029 (2)	−0.0104 (19)	0.0140 (18)	−0.0004 (18)
C7	0.029 (2)	0.044 (3)	0.036 (2)	−0.0053 (19)	0.0093 (19)	0.010 (2)
C8	0.039 (3)	0.054 (3)	0.033 (2)	−0.015 (2)	0.006 (2)	0.004 (2)
C9	0.046 (3)	0.050 (3)	0.023 (2)	−0.018 (2)	0.009 (2)	−0.006 (2)
C10	0.030 (2)	0.044 (2)	0.0231 (19)	−0.0135 (19)	0.0105 (18)	−0.0076 (18)
C11	0.031 (2)	0.038 (2)	0.0208 (19)	0.0041 (19)	0.0073 (17)	−0.0106 (17)
C12	0.025 (2)	0.0217 (19)	0.0210 (17)	−0.0046 (15)	0.0130 (16)	−0.0087 (14)
C13	0.0211 (19)	0.028 (2)	0.0150 (16)	0.0020 (15)	0.0061 (14)	−0.0025 (14)
C14	0.0150 (17)	0.0162 (16)	0.0195 (16)	−0.0015 (13)	0.0084 (14)	0.0015 (13)
C15	0.0176 (17)	0.0212 (18)	0.0208 (16)	0.0015 (14)	0.0107 (15)	0.0038 (14)
C16	0.028 (2)	0.031 (2)	0.0246 (18)	0.0110 (17)	0.0165 (17)	0.0087 (16)
C17	0.044 (3)	0.041 (2)	0.039 (2)	0.020 (2)	0.029 (2)	0.012 (2)
C18	0.034 (3)	0.053 (3)	0.050 (3)	0.025 (2)	0.024 (2)	0.018 (2)
C19	0.020 (2)	0.052 (3)	0.037 (2)	0.010 (2)	0.0046 (19)	0.012 (2)
C20	0.0206 (19)	0.031 (2)	0.0250 (18)	−0.0009 (16)	0.0075 (16)	0.0002 (17)
C21	0.032 (2)	0.043 (3)	0.030 (2)	0.0046 (19)	0.0197 (19)	0.0102 (19)
C22	0.030 (2)	0.0272 (19)	0.0199 (17)	−0.0069 (16)	0.0164 (17)	0.0021 (15)
C23	0.030 (2)	0.035 (2)	0.028 (2)	0.0038 (17)	0.0213 (18)	0.0071 (17)
C24	0.0179 (17)	0.0237 (19)	0.0191 (16)	−0.0057 (14)	0.0097 (14)	−0.0035 (14)
C25	0.0179 (18)	0.0194 (18)	0.0278 (18)	−0.0038 (14)	0.0139 (16)	−0.0030 (15)
C26	0.037 (2)	0.031 (2)	0.049 (3)	0.0023 (18)	0.033 (2)	0.0047 (19)
C27	0.042 (3)	0.030 (2)	0.069 (3)	0.0055 (19)	0.043 (3)	0.004 (2)
C28	0.026 (2)	0.026 (2)	0.059 (3)	0.0033 (17)	0.024 (2)	0.004 (2)
C29	0.025 (2)	0.032 (2)	0.037 (2)	0.0018 (17)	0.0169 (19)	0.0030 (18)
C30	0.0201 (19)	0.028 (2)	0.0292 (19)	−0.0006 (16)	0.0131 (16)	−0.0016 (17)
C31	0.028 (2)	0.0212 (19)	0.033 (2)	0.0055 (16)	0.0193 (18)	0.0017 (16)
C32	0.050 (3)	0.032 (2)	0.042 (3)	0.017 (2)	0.021 (2)	−0.001 (2)
C33	0.083 (4)	0.047 (3)	0.048 (3)	0.025 (3)	0.049 (3)	0.008 (2)
N1	0.0352 (19)	0.0240 (17)	0.0325 (17)	0.0077 (14)	0.0223 (16)	0.0009 (14)
O1	0.0303 (14)	0.0155 (12)	0.0202 (12)	−0.0092 (10)	0.0149 (11)	−0.0039 (10)
O2	0.0161 (13)	0.0287 (13)	0.0181 (12)	−0.0070 (10)	0.0072 (10)	−0.0047 (10)
O3	0.0205 (13)	0.0201 (12)	0.0169 (11)	0.0018 (10)	0.0073 (10)	−0.0016 (10)
O4	0.0180 (13)	0.0199 (12)	0.0192 (12)	0.0006 (10)	0.0092 (10)	−0.0031 (10)
O5	0.0250 (14)	0.0234 (13)	0.0221 (12)	−0.0002 (11)	0.0155 (11)	0.0030 (10)
O6	0.0188 (13)	0.0224 (13)	0.0199 (12)	0.0008 (10)	0.0130 (10)	0.0025 (10)
O7	0.0170 (13)	0.0179 (13)	0.0189 (12)	0.0002 (10)	0.0113 (10)	0.0013 (10)
O8	0.0284 (15)	0.0238 (13)	0.0259 (13)	−0.0015 (11)	0.0162 (12)	−0.0021 (11)
F1	0.075 (2)	0.0270 (13)	0.0293 (13)	0.0005 (12)	0.0248 (14)	−0.0080 (10)
F2	0.0503 (17)	0.0319 (13)	0.0557 (16)	−0.0103 (12)	0.0285 (14)	0.0114 (12)
F3	0.0451 (15)	0.0229 (12)	0.0314 (12)	−0.0004 (10)	0.0186 (12)	−0.0015 (10)
F4	0.0547 (17)	0.0318 (13)	0.0639 (18)	−0.0097 (12)	0.0380 (15)	−0.0225 (13)
F5	0.0423 (15)	0.0498 (16)	0.0424 (14)	−0.0010 (12)	0.0285 (13)	−0.0169 (12)
F6	0.066 (2)	0.072 (2)	0.0230 (13)	0.0296 (16)	0.0041 (13)	−0.0179 (13)
F7	0.0449 (16)	0.0691 (19)	0.0260 (13)	−0.0045 (14)	0.0138 (12)	0.0138 (12)
F8	0.0593 (18)	0.0378 (15)	0.0450 (15)	0.0059 (12)	0.0334 (14)	0.0191 (12)
F9	0.0599 (18)	0.0659 (18)	0.0527 (16)	0.0217 (14)	0.0480 (15)	0.0306 (14)
Hf1	0.01553 (8)	0.01823 (8)	0.01458 (7)	−0.00176 (6)	0.00772 (6)	−0.00041 (6)

### Geometric parameters (Å, °)

**Table:**

C1—F3	1.307 (5)	C21—F7	1.331 (5)
C1—F1	1.331 (4)	C21—F9	1.335 (4)
C1—F2	1.360 (4)	C21—C22	1.529 (5)
C1—C2	1.529 (5)	C22—O5	1.274 (4)
C2—O1	1.253 (4)	C22—C23	1.370 (5)
C2—C3	1.392 (5)	C23—C24	1.418 (5)
C3—C4	1.389 (5)	C23—H23	0.95
C3—H3	0.95	C24—O6	1.255 (4)
C4—O2	1.269 (4)	C24—C25	1.492 (5)
C4—C5	1.493 (5)	C25—C30	1.386 (5)
C5—C10	1.394 (5)	C25—C26	1.393 (5)
C5—C6	1.398 (6)	C26—C27	1.381 (6)
C6—C7	1.391 (6)	C26—H26	0.95
C6—H6	0.95	C27—C28	1.362 (6)
C7—C8	1.394 (7)	C27—H27	0.95
C7—H7	0.95	C28—C29	1.392 (6)
C8—C9	1.390 (7)	C28—H28	0.95
C8—H8	0.95	C29—C30	1.376 (5)
C9—C10	1.401 (6)	C29—H29	0.95
C9—H9	0.95	C30—H30	0.95
C10—H10	0.95	C31—O8	1.229 (4)
C11—F4	1.320 (5)	C31—N1	1.330 (4)
C11—F5	1.323 (5)	C31—H31	0.95
C11—F6	1.339 (4)	C32—N1	1.439 (5)
C11—C12	1.535 (5)	C32—H32A	0.98
C12—O3	1.279 (4)	C32—H32B	0.98
C12—C13	1.362 (5)	C32—H32C	0.98
C13—C14	1.404 (5)	C33—N1	1.451 (5)
C13—H13	0.95	C33—H33A	0.98
C14—O4	1.266 (4)	C33—H33B	0.98
C14—C15	1.484 (5)	C33—H33C	0.98
C15—C20	1.390 (5)	O1—Hf1	2.232 (2)
C15—C16	1.396 (5)	O2—Hf1	2.167 (2)
C16—C17	1.383 (5)	O3—Hf1	2.244 (2)
C16—H16	0.95	O4—Hf1	2.146 (2)
C17—C18	1.377 (6)	O5—Hf1	2.170 (2)
C17—H17	0.95	O6—Hf1	2.207 (2)
C18—C19	1.370 (6)	O7—Hf1	2.073 (2)
C18—H18	0.95	O7—Hf1^i^	2.135 (2)
C19—C20	1.379 (5)	O7—H1A	0.780 (19)
C19—H19	0.95	Hf1—O7^i^	2.135 (2)
C20—H20	0.95	Hf1—Hf1^i^	3.4958 (8)
C21—F8	1.327 (5)		
			
F3—C1—F1	108.7 (3)	C30—C25—C26	118.5 (3)
F3—C1—F2	107.3 (3)	C30—C25—C24	118.8 (3)
F1—C1—F2	106.2 (3)	C26—C25—C24	122.7 (3)
F3—C1—C2	113.6 (3)	C27—C26—C25	120.0 (4)
F1—C1—C2	112.5 (3)	C27—C26—H26	120
F2—C1—C2	108.2 (3)	C25—C26—H26	120
O1—C2—C3	127.7 (3)	C28—C27—C26	121.2 (4)
O1—C2—C1	113.4 (3)	C28—C27—H27	119.4
C3—C2—C1	118.6 (3)	C26—C27—H27	119.4
C4—C3—C2	120.5 (3)	C27—C28—C29	119.4 (4)
C4—C3—H3	119.8	C27—C28—H28	120.3
C2—C3—H3	119.8	C29—C28—H28	120.3
O2—C4—C3	123.0 (3)	C30—C29—C28	119.8 (4)
O2—C4—C5	115.6 (3)	C30—C29—H29	120.1
C3—C4—C5	121.2 (3)	C28—C29—H29	120.1
C10—C5—C6	119.2 (4)	C29—C30—C25	121.1 (4)
C10—C5—C4	121.7 (4)	C29—C30—H30	119.5
C6—C5—C4	119.1 (3)	C25—C30—H30	119.5
C7—C6—C5	121.3 (4)	O8—C31—N1	125.4 (4)
C7—C6—H6	119.3	O8—C31—H31	117.3
C5—C6—H6	119.3	N1—C31—H31	117.3
C6—C7—C8	119.2 (5)	N1—C32—H32A	109.5
C6—C7—H7	120.4	N1—C32—H32B	109.5
C8—C7—H7	120.4	H32A—C32—H32B	109.5
C9—C8—C7	120.0 (4)	N1—C32—H32C	109.5
C9—C8—H8	120	H32A—C32—H32C	109.5
C7—C8—H8	120	H32B—C32—H32C	109.5
C8—C9—C10	120.7 (4)	N1—C33—H33A	109.5
C8—C9—H9	119.6	N1—C33—H33B	109.5
C10—C9—H9	119.6	H33A—C33—H33B	109.5
C5—C10—C9	119.5 (4)	N1—C33—H33C	109.5
C5—C10—H10	120.2	H33A—C33—H33C	109.5
C9—C10—H10	120.2	H33B—C33—H33C	109.5
F4—C11—F5	106.8 (3)	C31—N1—C32	120.6 (3)
F4—C11—F6	107.0 (3)	C31—N1—C33	122.2 (3)
F5—C11—F6	107.0 (3)	C32—N1—C33	117.1 (3)
F4—C11—C12	111.3 (3)	C2—O1—Hf1	129.9 (2)
F5—C11—C12	111.7 (3)	C4—O2—Hf1	134.3 (2)
F6—C11—C12	112.7 (3)	C12—O3—Hf1	130.1 (2)
O3—C12—C13	128.8 (3)	C14—O4—Hf1	140.4 (2)
O3—C12—C11	112.5 (3)	C22—O5—Hf1	133.1 (2)
C13—C12—C11	118.7 (3)	C24—O6—Hf1	138.4 (2)
C12—C13—C14	120.1 (3)	Hf1—O7—Hf1^i^	112.34 (11)
C12—C13—H13	120	Hf1—O7—H1A	123 (3)
C14—C13—H13	120	Hf1^i^—O7—H1A	124 (3)
O4—C14—C13	121.2 (3)	O7—Hf1—O7^i^	67.66 (11)
O4—C14—C15	116.4 (3)	O7—Hf1—O4	107.84 (9)
C13—C14—C15	122.4 (3)	O7^i^—Hf1—O4	75.48 (9)
C20—C15—C16	119.1 (3)	O7—Hf1—O2	88.79 (9)
C20—C15—C14	122.9 (3)	O7^i^—Hf1—O2	79.46 (10)
C16—C15—C14	118.0 (3)	O4—Hf1—O2	141.33 (9)
C17—C16—C15	120.2 (4)	O7—Hf1—O5	145.64 (9)
C17—C16—H16	119.9	O7^i^—Hf1—O5	146.39 (9)
C15—C16—H16	119.9	O4—Hf1—O5	85.28 (9)
C18—C17—C16	119.4 (4)	O2—Hf1—O5	100.53 (9)
C18—C17—H17	120.3	O7—Hf1—O6	76.60 (9)
C16—C17—H17	120.3	O7^i^—Hf1—O6	133.34 (9)
C19—C18—C17	121.1 (4)	O4—Hf1—O6	146.29 (9)
C19—C18—H18	119.5	O2—Hf1—O6	70.56 (9)
C17—C18—H18	119.5	O5—Hf1—O6	75.60 (9)
C18—C19—C20	119.9 (4)	O7—Hf1—O1	143.71 (9)
C18—C19—H19	120.1	O7^i^—Hf1—O1	77.53 (9)
C20—C19—H19	120.1	O4—Hf1—O1	71.51 (9)
C19—C20—C15	120.2 (4)	O2—Hf1—O1	74.67 (9)
C19—C20—H20	119.9	O5—Hf1—O1	70.26 (9)
C15—C20—H20	119.9	O6—Hf1—O1	124.85 (9)
F8—C21—F7	107.1 (3)	O7—Hf1—O3	78.55 (9)
F8—C21—F9	106.7 (3)	O7^i^—Hf1—O3	123.38 (9)
F7—C21—F9	107.3 (3)	O4—Hf1—O3	73.49 (9)
F8—C21—C22	112.0 (3)	O2—Hf1—O3	145.08 (9)
F7—C21—C22	110.0 (3)	O5—Hf1—O3	75.02 (9)
F9—C21—C22	113.4 (3)	O6—Hf1—O3	74.86 (9)
O5—C22—C23	128.6 (3)	O1—Hf1—O3	131.92 (8)
O5—C22—C21	112.1 (3)	O7—Hf1—Hf1^i^	34.39 (7)
C23—C22—C21	119.3 (3)	O7^i^—Hf1—Hf1^i^	33.27 (6)
C22—C23—C24	120.9 (3)	O4—Hf1—Hf1^i^	91.64 (7)
C22—C23—H23	119.5	O2—Hf1—Hf1^i^	82.86 (7)
C24—C23—H23	119.5	O5—Hf1—Hf1^i^	176.54 (7)
O6—C24—C23	121.8 (3)	O6—Hf1—Hf1^i^	106.36 (6)
O6—C24—C25	117.0 (3)	O1—Hf1—Hf1^i^	110.26 (6)
C23—C24—C25	121.2 (3)	O3—Hf1—Hf1^i^	102.61 (6)
			
F3—C1—C2—O1	31.8 (4)	O8—C31—N1—C33	−178.3 (4)
F1—C1—C2—O1	155.8 (3)	C3—C2—O1—Hf1	15.9 (5)
F2—C1—C2—O1	−87.2 (4)	C1—C2—O1—Hf1	−170.8 (2)
F3—C1—C2—C3	−154.2 (3)	C3—C4—O2—Hf1	−24.7 (5)
F1—C1—C2—C3	−30.2 (5)	C5—C4—O2—Hf1	159.6 (2)
F2—C1—C2—C3	86.8 (4)	C13—C12—O3—Hf1	−18.8 (6)
O1—C2—C3—C4	9.1 (6)	C11—C12—O3—Hf1	162.2 (2)
C1—C2—C3—C4	−163.9 (3)	C13—C14—O4—Hf1	13.5 (6)
C2—C3—C4—O2	−5.8 (5)	C15—C14—O4—Hf1	−166.8 (2)
C2—C3—C4—C5	169.7 (3)	C23—C22—O5—Hf1	−13.5 (6)
O2—C4—C5—C10	−168.6 (3)	C21—C22—O5—Hf1	165.4 (2)
C3—C4—C5—C10	15.6 (5)	C23—C24—O6—Hf1	0.2 (5)
O2—C4—C5—C6	11.8 (5)	C25—C24—O6—Hf1	179.5 (2)
C3—C4—C5—C6	−164.0 (3)	Hf1^i^—O7—Hf1—O7^i^	0
C10—C5—C6—C7	−1.3 (6)	Hf1^i^—O7—Hf1—O4	−65.36 (13)
C4—C5—C6—C7	178.3 (3)	Hf1^i^—O7—Hf1—O2	79.09 (12)
C5—C6—C7—C8	2.2 (6)	Hf1^i^—O7—Hf1—O5	−173.87 (12)
C6—C7—C8—C9	−1.0 (7)	Hf1^i^—O7—Hf1—O6	149.39 (13)
C7—C8—C9—C10	−1.1 (7)	Hf1^i^—O7—Hf1—O1	17.5 (2)
C6—C5—C10—C9	−0.8 (6)	Hf1^i^—O7—Hf1—O3	−133.65 (12)
C4—C5—C10—C9	179.6 (4)	C14—O4—Hf1—O7	−95.4 (4)
C8—C9—C10—C5	2.0 (6)	C14—O4—Hf1—O7^i^	−155.6 (4)
F4—C11—C12—O3	65.1 (4)	C14—O4—Hf1—O2	153.1 (3)
F5—C11—C12—O3	−54.1 (5)	C14—O4—Hf1—O5	52.2 (3)
F6—C11—C12—O3	−174.6 (3)	C14—O4—Hf1—O6	−2.8 (4)
F4—C11—C12—C13	−114.0 (4)	C14—O4—Hf1—O1	122.9 (4)
F5—C11—C12—C13	126.8 (4)	C14—O4—Hf1—O3	−23.6 (3)
F6—C11—C12—C13	6.2 (6)	C14—O4—Hf1—Hf1^i^	−126.3 (3)
O3—C12—C13—C14	−3.6 (6)	C4—O2—Hf1—O7	−113.9 (3)
C11—C12—C13—C14	175.4 (3)	C4—O2—Hf1—O7^i^	−46.4 (3)
C12—C13—C14—O4	7.8 (5)	C4—O2—Hf1—O4	3.8 (4)
C12—C13—C14—C15	−172.0 (3)	C4—O2—Hf1—O5	99.4 (3)
O4—C14—C15—C20	−172.3 (3)	C4—O2—Hf1—O6	169.9 (3)
C13—C14—C15—C20	7.4 (5)	C4—O2—Hf1—O1	33.5 (3)
O4—C14—C15—C16	8.0 (5)	C4—O2—Hf1—O3	178.3 (3)
C13—C14—C15—C16	−172.3 (3)	C4—O2—Hf1—Hf1^i^	−79.9 (3)
C20—C15—C16—C17	0.3 (5)	C22—O5—Hf1—O7	−23.2 (4)
C14—C15—C16—C17	180.0 (3)	C22—O5—Hf1—O7^i^	167.1 (3)
C15—C16—C17—C18	1.2 (6)	C22—O5—Hf1—O4	−138.3 (3)
C16—C17—C18—C19	−2.4 (7)	C22—O5—Hf1—O2	80.3 (3)
C17—C18—C19—C20	2.0 (7)	C22—O5—Hf1—O6	13.7 (3)
C18—C19—C20—C15	−0.5 (7)	C22—O5—Hf1—O1	149.7 (3)
C16—C15—C20—C19	−0.7 (6)	C22—O5—Hf1—O3	−64.1 (3)
C14—C15—C20—C19	179.7 (4)	C24—O6—Hf1—O7	151.9 (3)
F8—C21—C22—O5	52.0 (4)	C24—O6—Hf1—O7^i^	−167.7 (3)
F7—C21—C22—O5	−67.0 (4)	C24—O6—Hf1—O4	49.8 (4)
F9—C21—C22—O5	172.8 (3)	C24—O6—Hf1—O2	−114.6 (3)
F8—C21—C22—C23	−129.0 (4)	C24—O6—Hf1—O5	−7.7 (3)
F7—C21—C22—C23	112.0 (4)	C24—O6—Hf1—O1	−60.5 (3)
F9—C21—C22—C23	−8.2 (5)	C24—O6—Hf1—O3	70.4 (3)
O5—C22—C23—C24	−0.2 (7)	C24—O6—Hf1—Hf1^i^	169.4 (3)
C21—C22—C23—C24	−179.0 (3)	C2—O1—Hf1—O7	38.1 (4)
C22—C23—C24—O6	6.7 (6)	C2—O1—Hf1—O7^i^	54.6 (3)
C22—C23—C24—C25	−172.7 (3)	C2—O1—Hf1—O4	133.2 (3)
O6—C24—C25—C30	−6.3 (5)	C2—O1—Hf1—O2	−27.7 (3)
C23—C24—C25—C30	173.0 (3)	C2—O1—Hf1—O5	−135.2 (3)
O6—C24—C25—C26	173.7 (3)	C2—O1—Hf1—O6	−80.1 (3)
C23—C24—C25—C26	−6.9 (5)	C2—O1—Hf1—O3	178.6 (3)
C30—C25—C26—C27	0.0 (6)	C2—O1—Hf1—Hf1^i^	48.5 (3)
C24—C25—C26—C27	−180.0 (4)	C12—O3—Hf1—O7	136.6 (3)
C25—C26—C27—C28	0.8 (7)	C12—O3—Hf1—O7^i^	83.4 (3)
C26—C27—C28—C29	−1.2 (7)	C12—O3—Hf1—O4	23.9 (3)
C27—C28—C29—C30	0.9 (6)	C12—O3—Hf1—O2	−152.5 (3)
C28—C29—C30—C25	−0.1 (6)	C12—O3—Hf1—O5	−65.5 (3)
C26—C25—C30—C29	−0.4 (6)	C12—O3—Hf1—O6	−144.3 (3)
C24—C25—C30—C29	179.7 (3)	C12—O3—Hf1—O1	−20.8 (3)
O8—C31—N1—C32	−2.1 (6)	C12—O3—Hf1—Hf1^i^	111.9 (3)

Symmetry codes: (i) −*x*+1, −*y*+1, −*z*+1.

### Hydrogen-bond geometry (Å, °)

**Table:**

*D*—H···*A*	*D*—H	H···*A*	*D*···*A*	*D*—H···*A*
O7—H1A···O8	0.78 (2)	1.94 (2)	2.712 (3)	171 (5)
